# Plant-Derived *Lactobacillus paracasei* IJH-SONE68 Improves Chronic Allergy Status: A Randomized, Double-Blind, Placebo-Controlled Clinical Trial

**DOI:** 10.3390/nu13114022

**Published:** 2021-11-11

**Authors:** Masafumi Noda, Keishi Kanno, Narandalai Danshiitsoodol, Fumiko Higashikawa, Masanori Sugiyama

**Affiliations:** 1 Department of Probiotic Science for Preventive Medicine, Graduate School of Biomedical and Health Sciences, Hiroshima University, Kasumi 1–2–3, Minami-ku, Hiroshima 734-8551, Japan; bel@hiroshima-u.ac.jp (M.N.); naraa@hiroshima-u.ac.jp (N.D.); fumiko@hiroshima-u.ac.jp (F.H.); 2 Department of General Internal Medicine, Hiroshima University Hospital, Kasumi 1–2–3, Minami-Ku, Hiroshima 734-8551, Japan; kkanno@hiroshima-u.ac.jp; 3 Department of Clinical Pharmaceutical and Therapeutics, Hiroshima University, Kasumi 1–2–3, Minami-ku, Hiroshima 734-8551, Japan

**Keywords:** *Lactobacillus paracasei*, chronic allergy, clinical trial, plant-derived lactic acid bacterium, anti-inflammation

## Abstract

We have recently demonstrated that the exopolysaccharides (EPSs) produced by a plant-derived lactic acid bacterium, *Lactobacillus paracasei* IJH-SONE68, prevent and ameliorate allergic reaction on contact in dermatitis model mice. In the present study, we conducted a clinical trial using a capsule containing spray-dried powder from pineapple juice broth fermented with the LAB strain as an experimental diet. The clinical trial was conducted as a double-blind and placebo-controlled randomized comparative study from May 2019 to July 2021. Males and females between the ages of 21 and 70 who experience chronic allergies participated in the study. Sixty subjects were instructed to orally take a capsule containing the IJH-SONE68 powder or placebo, every day for 12 weeks. After the clinical trial was over, the scores based on subjects’ self-assessment of allergic status were significantly improved in the intervention group, as compared with the placebo group. Some serum biochemicals associated with inflammation response were also significantly improved by intake of the experimental diet. In conclusion, the IJH-SONE68-derived EPS improves chronic allergy status in humans and is expected to decrease their inconvenience.

## 1. Introduction

Allergic diseases are immunoglobulin E (IgE)-mediated type I inflammatory disorders, including allergic rhinitis [1,2]. The major symptoms of allergic rhinitis are sneezing, nasal congestion, and lacrimation caused by complex allergen-driven mucosal inflammations, and the increased inflammatory mediators lead to sensory nerve activation, plasma leakage, and congestion [3,4]. The prevalence of these diseases in the USA ranges from 9 to 42%, and in the UK, it reaches 26% in adults [1,5]. An epidemiological study in Japan revealed that the prevalence of allergic rhinitis markedly increased from 29.8 to 39.4%, between 1998 and 2008 [6]. Allergic rhinitis is manageable with appropriate treatment but resists cure; thus, the treatment is aimed at alleviating symptoms and removing difficulties to improve patients’ quality of life (QOL) [6].

Lactic acid bacteria (LABs) is a generic name given to Gram-positive bacteria that produce a large amount of lactic acid [7]. The LABs are generally non-pathogenic and many strains of them produce functional substances that have health benefits for humans [8,9]. Therefore, some beneficial LAB strains are regarded as probiotics, which are defined as, living microorganisms conferring health benefits to hosts when consumed in adequate amounts [10].

We have isolated many kinds of LABs from natural sources, especially from raw plants, including fruits and medicinal herbs. Through randomized clinical trials, we have found in our established plant-derived LAB library of more than 1000 strains, some useful ones for preventive medicine, such as for improving constipation, obesity, and hepatic dysfunction [11,12,13]. Furthermore, we have confirmed that some of the isolates produce exopolysaccharides (EPSs) outside cells [14,15,16,17]. Interestingly, EPSs have been found to inhibit the catalytic activity of hyaluronidase (EC 3.2.1.35), which catalyzes the hydrolysis of the main extracellular matrix constituent, hyaluronic acid. Under inflammatory conditions, the hydrolysis of hyaluronic acid makes inflammation worse [18,19,20,21]. The inhibitory phenomenon against hyaluronidase, as some anti-allergic agents, correlates with the inhibition of IgE-mediated histamine release from mast cells during inflammatory reactions [22,23].

One of the EPS-producing strains, *Lactobacillus* (*Lb.*) *paracasei* IJH-SONE68, was isolated from a fig leaf. Our previous study revealed that the IJH-SONE68-derived neutral EPS has a novel structure, which mainly consists of α-1,6-linked chains made of *N*-acetylglucosamine [17]. In addition, the IJH-SONE68-derived EPSs inhibit the catalytic activity of hyaluronidase, as well as some commercially used anti-allergic and anti-inflammation agents [15,17,24,25]. Our recent animal experiments also showed that IJH-SONE68-derived EPSs prevent and improve picryl chloride-induced contact dermatitis and dextran sulfate sodium (DSS)-induced ulcerative colitis on model mice, by repressing the accelerated expression of interleukin (IL)-4 and the serum IgE level [26] and by reducing the MIP-2 expression [27], respectively. Therefore, in the present study, we aim to show that IJH-SONE68-derived EPSs improve allergic conditions by conducting a clinical trial involving subjects with perennial allergy symptoms, which may be accompanied by chronic inflammation.

## 2. Materials and Methods

### 2.1. Subjects 

**Figure 1:**
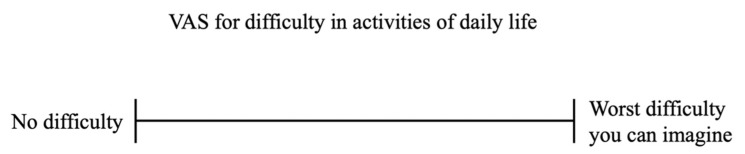
VAS for difficulty in daily life activities for self-assessment questionnaire (100 mm width, without scale indication).

Healthy volunteers who live in the Hiroshima area of Japan and who are between 20 and 70 years old, were recruited through a series of advertisements. The inclusion criteria were that subjects must be healthy males or females who have at least once (1) a score of 8 to 20 on the self-assessment questionnaire for allergic symptoms (Table 1) and (2) a score of 40 or above for the visual analogue scale (VAS) value, indicating difficulty in daily life activities (Figure 1). The items and scores were designed according to the criteria previously developed in guidelines [6,28]. The exclusion criteria were applied to volunteers who (1) have allergic hypersensitivity to pineapple; (2) use regular medication; (3) are pregnant or breastfeeding; (4) take medicines, supplements, or functional foods that may affect allergic symptoms; or (5) participated in any other clinical trials within 3 months of the commencement of this study. Written informed consent was obtained from each participant before the start of the trial.

### 2.2. Study Capsules and Placebo

We delegated the production of placebo and test capsules to SAKURAO Brewery and Distillery Co., Ltd., Japan. The test capsules contained heat-killed IJH-SONE68-strain powder and dextrin, whereas the placebo capsules contained dextrin only. The IJH-SONE68 strain was cultured in pineapple juice and then the cultured broth was heat-treated before applying a spray dryer. In total, 1040 mg of IJH-SONE68 powder, which contained approximately 5.0 × 10^5^ cells, was used to fill four separate capsules. The placebo capsule contained the same amount of dextrin.

### 2.3. Study Design

This trial was conducted at Hiroshima University (Hiroshima, Japan) from May 2019 to July 2021 using a double-blind, randomized, placebo-controlled parallel-group study. Eligible subjects who met the criteria were enrolled and stratified according to their score on the self-assessment questionnaire (12 ≤ or less) and serum IgE concentration (251 ≤ IU/mL or less), then assigned to the IJH-SONE68 or placebo group using the blocked randomization method in a 1:1 allocation ratio with a block size of 4. The allocation table was generated using Microsoft Excel software. Randomization assignments were carried out by non-clinical staff, with no analytical involvement in the present trial. The subjects and outcome assessors were blinded to the assignment information.

The subjects were instructed to take 4 capsules of either the IJH-SONE68 or placebo daily at any time within the day for 12 weeks. They were also directed to keep their ordinary dietary habitats and not to donate blood during the trial period. The subjects were provided with daily dated record forms throughout the trial period, to make a record of their consumption of capsules and their health conditions. The forms also contained a daily answer sheet for a self-assessment questionnaire and VAS value. Adverse events that newly emerged or worsened after intervention were evaluated when those grades shifted higher, according to the Common Terminology Criteria for Adverse Events version 5.0 (CTCAE v5.0).

The primary outcomes of the present study were, changes in the self-assessment questionnaire and VAS value, and the secondary outcomes were, changes in the serum total IgE and specific IgE (for 2 types of house dust, Gramineae pollen, weed pollen, animal dander, and fungi) levels. The subjects visited Hiroshima University for physical examinations (every 4 weeks), serum total IgE measurements (every 4 weeks), answering the self-assessment questionnaire and assigning a VAS value (every 4 weeks), and serum specific IgE and biochemical measurements and urinalysis (at 12 weeks). Blood samples were obtained after a 9 h overnight fast. Body fat percentage was measured using a body composition analyzer (BC-118E, Tanita, Tokyo, Japan). Blood pressure was measured according to the Japanese Society of Hypertension Guidelines for the Management of Hypertension 2019 (JSH2019) [29].

The protocol of the present clinical trial was approved by the Ethics Committee of Hiroshima University (approval no. C-266) and performed according to the guidelines of the Helsinki Declaration. This trial was registered in the University Hospital Medical Information Network Clinical Trials Registry (UMIN-CTR), ID: UMIN000036317, on 11 March 2019.

### 2.4. Statistical Analysis

The sample size in each group in the present study was calculated as 46, to detect 10% difference with an estimated S.D. of 20% for total IgE, assuming 80% power and a two-sided significance level of 0.05 using two-sample *t*-test. The difference and S.D. for sample size estimation were performed on the basis of the data from our previous animal study [26]. The obtained data were analyzed according to the intention-to-treat principle, and the multiple-imputation method was applied to missing data [30]. For each outcome, 20 multiple-imputed data sets were generated and the resultant analyses were combined. The analysis of covariance (ANCOVA) was applied to changes from the baseline for primary outcomes and serum total IgE using each baseline value as a covariate. The baseline characteristics were compared using the unpaired Student’s *t*-test. Statistical analysis for changes in each item on the questionnaire was done using the Mann–Whitney U test, since the results did not show a normal distribution. All statistical analyses were performed using IBM SPSS Statistics 17.0 J for Windows (IBM Japan, Tokyo, Japan).

## 3. Results

### 3.1. Recruitment of Subjects

**Figure 2:**
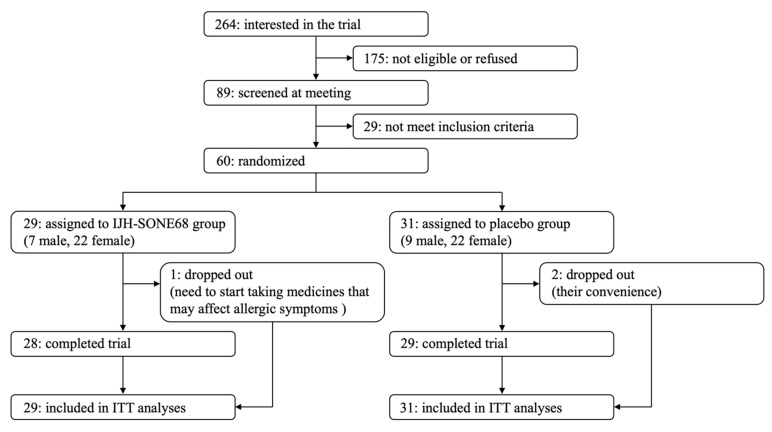
Flow diagram of subjects in the study.

Recruitment for the present trial is summarized in Figure 2. Among 264 applicants who were interested in the trial, 175 did not satisfy the inclusion criteria or canceled their application. After participating in the explanatory meeting, 60 eligible subjects (aged 21–70) were enrolled in the study and were randomly divided into two groups, the IJH-SONE68-intake group or the placebo group.

Based on the scheduled deadline and the limited financial resources, it was difficult to recruit subjects in a short period of time with prevention of the spread of COVID-19. Therefore, this study was unavoidably completed before the original registration goal was achieved (*n* = 100). The baseline characteristics of the subjects are summarized in Table 2. Between two groups, there were no significant differences in the listed items. During the trial period, 3 subjects dropped out from the study because of the need to start taking medicines that may affect allergic symptoms (1 in the IJH-SONE68 group) and their convenience (2 in the placebo group). The remaining 57 subjects completed the study (95% rate). The data obtained from all subjects were analyzed after filling in the missing data by the multiple imputation method. The average compliance rates for taking capsules daily were 94.9% and 95.6% in the IJH-SONE68 and placebo groups, respectively. The blinding success confirmed by the questionnaire resulted in 35.0% correct, 23.3% incorrect, and 40.0% “cannot judge” answers.

### 3.2. Effects of IJH-SONE68 on Primary Outcomes

The changes in primary outcomes, which were the self-assessment questionnaire scores and the VAS values (Table 1 and Figure 1), during the trial period are summarized in Table 3. Although the average self-assessment questionnaire score in both groups decreased, there was a significant difference between the changes in questionnaire scores (*p* = 0.008). Likewise, the average VAS value in both groups decreased considerably, but changes in the value were not significant (*p* = 0.380).

The score changes in each item of the self-assessment questionnaire were also compared (Table 4). Although the average scores for each of the six items decreased in both groups, the scores for head dullness, watery eyes, and frequency of nose-blowing and sneezing in the IJH-SONE68 group decreased, more than those in the placebo group (*p* < 0.05).

### 3.3. Effects of IJH-SONE68 on Secondary Outcomes

**Figure 3:**
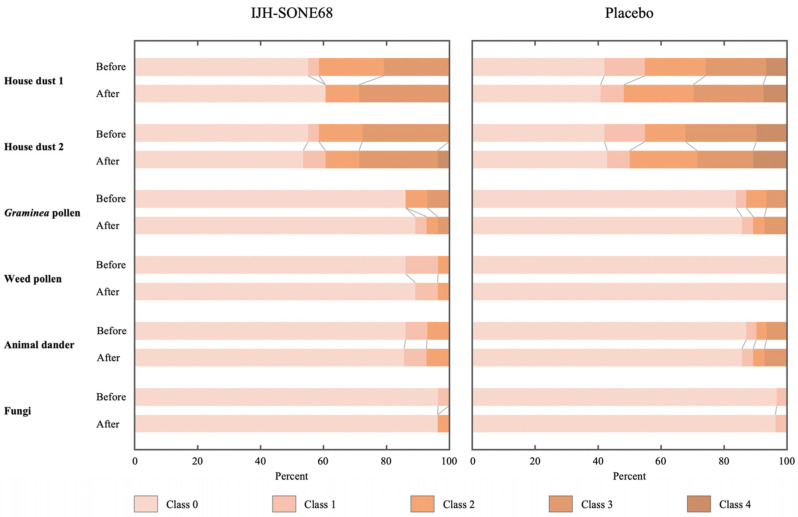
Stacked bar plot describing the specific IgE classifications of subjects for each of the 6 allergens before and after treatment.

The serum total IgE decreased slightly in the IJH-SONE68 group; however, there was no significant difference between the two groups for changes in the total IgE (Table 5). Although the decrease in serum specific IgE was also assessed as a secondary outcome, there were not enough subjects who were positive for each of the six specific IgE values, according to the semi-quantitative classification (Table 6 and Figure 3).

### 3.4. Monitoring the Adverse Effects

Physical examinations and other serum parameters were also monitored to detect adverse effects of IJH-SONE68 intake. According to the CTCAE v5.0, there were no significant differences in adverse events possibly related to the study design, between the IJH-SONE68 and placebo groups (Table 7). In addition, in the IJH-SONE68 group, the average parameters for serum aspartate aminotransferase (AST), alanine aminotransferase (ALT), alkaline phosphatase (ALP), and cholinesterase (ChE), were decreased from the baseline, and the changes in those parameters were significant when compared with the placebo group (Supplementary Table S1). No abnormal change was observed in the urinalysis data throughout this clinical trial.

## 4. Discussion

**Figure 4:**
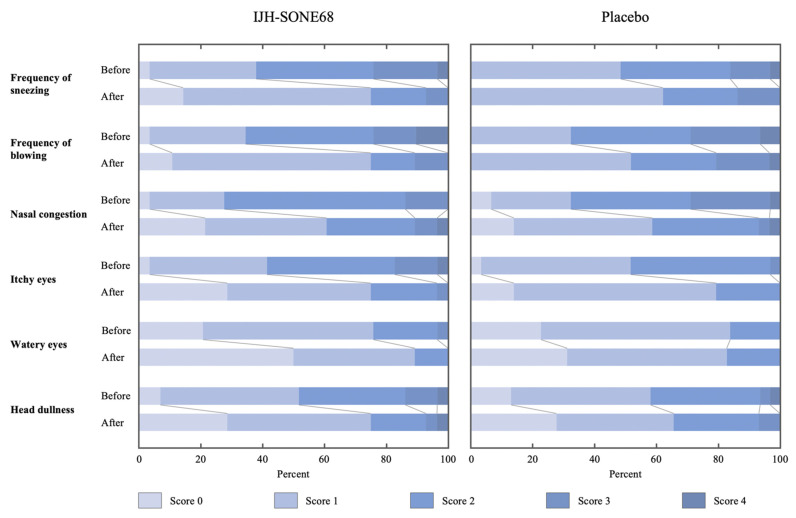
Stacked bar plot describing the scores provided by subjects in the self-assessment questionnaire for each of the 6 items before and after treatment.

Different from seasonal allergies like pollinosis, which is caused by pollen allergens, perennial allergies cause chronic symptoms that appear year-round or intermittently. Therefore, perennial allergies considerably decrease the QOL for those patients. Common allergens that account for perennial allergy symptoms are indoor allergens, such as house dust mites (mostly), animal dander, cockroaches, and fungi [31,32]. A number of symptoms, including head dullness, nasal congestion, runny nose, watery and itchy eyes, and sneezing, are expected to be present in a person who has perennial allergies as well as in those with seasonal allergies. In the present trial, the self-assessment questionnaire answers regarding those symptoms, are scored and analyzed as primary outcomes. As shown in Table 4, the results indicate that the IJH-SONE68 capsule significantly improved some of the symptoms. More detailed changes in the composition of questionnaire scores before and after the intake of IJH-SONE68 or placebo capsules, are shown in Figure 4. Regardless of group, the questionnaire scores decreased for each item throughout the 12 weeks of treatment, especially for frequency of nose blowing and sneezing. On the other hand, no significant differences were observed between the groups in the other primary outcome, the VAS value, indicating that the value might reflect other minor factors in addition to the six items asked in the questionnaire.

Previously, we showed that the IJH-SONE68-derived EPSs significantly decrease the accelerated serum IgE level and the expression of IL-4 mRNA, in the contact dermatitis model mice [26]. As a preliminary experiment, we also confirmed the improving effect of the IJH-SONE68 strain in the animal model of immediate hypersensitivity, using active cutaneous anaphylaxis model mice [33,34]. Allergic rhinitis is one of the common allergic disorders, which is classified as an immediate allergy [6]. In this allergy type, the allergens promote the proliferation of T helper 2 (Th2) cell, resulting in IL-4 release, followed by the antigen-specific IgE production. However, no significant difference was observed in the changes in serum total IgE levels between the groups. Although the serum total IgE was measured as a secondary outcome of the result of the previous animal studies [26,34], the baseline serum IgE levels ranged from 5 to 1410 IU/mL in the subjects, resulting in a biased population structure and biased result. When the subjects with lower IgE levels (less than 251 IU/mL) were analyzed using the Mann–Whitney U test, there was a trend difference between the two groups for changes in total IgE (*p* = 0.077), indicating that the result partially corresponds to data obtained from the animal experiment [26].

Our recent study also showed that the IJH-SONE68-derived EPS prevents and ameliorates inflammatory responses in the DSS-induced ulcerative colitis model mice with significant repression of MIP-2, which is a functional analogue of human IL-8 [27]. The inflammatory cytokine IL-8 has been reported to play an important role during chronic disease progression through binding to IL-8 receptors A and B, located on the neutrophils [35,36,37]. Although the changes of cytokines were not analyzed in the present study, allergic symptoms of some subjects with high IgE levels (251 IU/mL or more) might become chronic disease. The precise mechanisms of the IJH-SONE68-derived EPS are not clear yet. However, the EPS seems to modulate excessive immune responses, judging from the result obtained by animal experiments [26,27]. Therefore, by affecting not only IgE-mediated allergic reaction, but also chronic inflammation, the IJH-SONE68-derived EPS might improve the self-assessment questionnaire in the subjects.

There were only a few subjects with specific IgE-positive results; therefore, a statistical analysis could not be conducted. As shown in Figure 3, the composition of the specific IgE classification of each item was basically unchanged throughout the trial period. Considering the decreasing trend observed in the lower IgE group, IJH-SONE68 capsule intake might ameliorate allergic responses, nonspecifically.

The amelioration of the self-assessment questionnaire scores seems to be due to the anti-inflammatory effect of the IJH-SONE68-derived EPSs confirmed in previous studies [26,31,33], in addition to the decrease in IgE level. Although other biochemical measurements, which were monitored to detect adverse effects of IJH-SONE68 intake, were neither primary nor secondary outcomes, serum levels of AST, ALT, ALP, and ChE were significantly improved in the IJH-SONE68 group. AST and ALT catalyze the transamination reaction to generate oxaloacetic acid and pyruvic acids, respectively, which are necessary to run a citric acid cycle. Both enzymes are highly concentrated in the liver, but AST is also widely distributed in the heart, muscles, and kidneys [38]. Therefore, both elevated serum levels reflect cell injury, but the ALT level is more specific to liver damage. ALP is also present in many mammalian tissues and its elevation in serum is correlated with tissue injury, specifically in liver diseases [39]. As described above, those enzymes leak into the blood stream under inflammatory conditions; thus, the IJH-SONE68-derived EPSs might partially ameliorate those serum biochemical parameters. Since those parameters were not paid attention to and were not assigned as inclusion criteria, further trials with suitable subjects will be needed for clarification.

ChE catalyzes the hydrolyzation of choline esters like acetylcholine and the enzyme is synthesized mainly in hepatocytes. A decrease in the ChE level is observed in patients with liver dysfunction, such as cirrhosis; whereas a high serum ChE level has been reported to be associated with obesity and metabolic syndrome [40,41,42]. Interestingly, the trend difference between the changes in body fat percentage of the two groups, was observed (*p* = 0.058). Our preliminary study suggested that IJH-SONE68 also has an anti-obesity effect, and therefore, lipid metabolism and fatty liver might be improved during the trial, resulting in the decrease of ALT and ChE. The present study indicated that the IJH-SONE68 strain can be expected to help persons with perennial allergies, to decrease their inconvenience.

## 5. Conclusions

The oral administration of the spray-dried powder derived from the culture broth of *Lb. paracasei* IJH-SONE68, has been shown to significantly improve the scores based on subjects’ self-assessment of allergic status in the present clinical study. The present outcome demonstrates that the IJH-SONE68 strain can be expected to help persons with perennial allergies and decrease inconvenience.

## Figures and Tables

**Table 1:** Questionnaire items for self-assessment and scoring characteristics.

Score	Items					
	Frequency of Sneezing (Average Times/Day)	Frequency of Blowing (Average Times/Day)	Nasal Congestion	Itchy Eyes	Watery Eyes	Head Dullness
0	No sneezing	No sneezing	None of the time	None of the time	None of the time	None of the time
1	1–5	1–5	A little of the time (without mouth-breathing)	Mild	Mild	Mild
2	6–10	6–10	Some of the time (with mouth-breathing)	Moderate to severe	Moderate to severe	Moderate to severe
3	11–20	11–20	Most of the time (with mouth-breathing)	Severe	Severe	Severe
4	21 or more	21 or more	All of the time (with mouth-breathing)	Very severe	Very severe	Very severe

**Table 2:** Baseline characteristics of the subjects.

	IJH-SONE68 (*n* = 29)	Placebo (*n* = 31)	*p* Value
Age (years)	51.6 ± 14.4	54.8 ± 10.5	0.331
Male	53.0 ± 17.4 (*n* = 7)	56.3 ± 12.0 (*n* = 9)	0.674
Female	51.1 ± 13.8 (*n* = 22)	54.2 ± 10.1 (*n* = 22)	0.408
Height (cm)	160.1 ± 9.6	160.0 ± 7.7	0.834
Body weight (kg)	55.4 ± 15.6	56.4 ± 12.4	0.796
Systolic blood pressure (mmHg)	110.1 ± 15.5	118.6 ± 16.9	0.067
Diastolic blood pressure (mmHg)	67.8 ± 11.2	73.5 ± 12.3	0.067

Data are indicated as mean ± S.D. *p* values are calculated using unpaired Student’s *t*-test.

**Table 3:** Changes in primary outcomes in the study.

	IJH-SONE68 (*n* = 29)	Placebo (*n* = 31)	*p* Value
Summary of self-questionnaire score			0.008
Baseline	10.1 ± 0.6	9.5 ± 0.3	
Change at 12 week	−3.6 ± 0.5	−1.9 ± 0.5	
VAS value			0.380
Baseline	52.3 ± 2.7	57.4 ± 3.4	
Change at 12 week	−17.6 ± 3.6	−13.1 ± 3.5	

Data are indicated as mean ± S.E. *p* values between IJH-SONE68 and placebo groups are calculated by ANCOVA using each baseline value as a covariate.

**Table 4:** Changes in each item of the self-assessment questionnaire.

		IJH-SONE68 (*n* = 29)	Placebo (*n* = 31)	*p* Value
Frequency of sneezing				<0.05
	Baseline	1.9 ± 0.2	1.7 ± 0.2	
	12 week	1.2 ± 0.2	1.5 ± 0.1	
Frequency of blowing				<0.05
	Baseline	2.0 ± 0.2	2.0 ± 0.2	
	12 week	1.3 ± 0.2	1.7 ± 0.2	
Nasal congestion				-
	Baseline	1.8 ± 0.1	1.9 ± 0.2	
	12 week	1.3 ± 0.2	1.4 ± 0.2	
Itchy eyes				-
	Baseline	1.8 ± 0.2	1.5 ± 0.1	
	12 week	1.0 ± 0.2	1.1 ± 0.1	
Watery eyes				<0.05
	Baseline	1.1 ± 0.1	0.9 ± 0.1	
	12 week	0.6 ± 0.1	0.9 ± 0.1	
Head dullness				<0.05
	Baseline	1.6 ± 0.2	1.4 ± 0.2	
	12 week	1.1 ± 0.2	1.1 ± 0.2	

Data are indicated as mean ± S.E. *p* values between IJH-SONE68 and placebo groups are calculated by the Mann–Whitney *U* test.

**Table 5:** Changes in serum total IgE in the study.

	IJH-SONE68 (*n* = 29)	Placebo (*n* = 31)	*p* Value
Total IgE (log_10_ IU/mL)			0.361
Baseline	1.87 ± 0.10	1.90 ± 0.10	
Change at 12 week	−0.004 ± 0.019	0.021 ± 0.019	

Data are indicated as mean ± S.E. *p* values between IJH-SONE68 and placebo groups are calculated by ANCOVA using each baseline value as a covariate.

**Table 6:** Classification of specific IgE levels.

Class	Specific IgE Titer (U_A_/mL)	Quantitative Analysis
0	<0.35	Negative
1	0.35 to <0.70	Boundary
2	0.70 to <3.50	Positive
3	3.50 to <17.5	Positive
4	17.5 to <50.0	Positive
5	50.0 to <100	Positive
6	100<	Positive

**Table 7:** Number of subjects who show adverse events that were possibly related to the study design or treatments.

		IJH-SONE68 (*n* = 29)	Placebo (*n* = 31)	*p* Value
Anemia				0.229
	Grade 1	2 (7%)	0	
Blood bilirubin increased				0.942
	Grade 1	0	2 (6%)	
Blood lactate dehydrogenase increased				0.355
	Grade 1	1 (3%)	4 (13%)	
Cholesterol high				0.518
	Grade 1	12 (41%)	16 (52%)	
	Grade 2	1 (3%)	0	
Creatinine increased				1.000
	Grade 1	2 (7%)	2 (6%)	
Weight gain				0.737
	Grade 1	0	1 (3%)	
	Grade 2	1 (3%)	0	
White blood cell decreased				0.666
	Grade 1	3 (10%)	2 (6%)	
Hyperglycemia				0.148
	Grade 1	2 (7%)	7 (23%)	
Hypertriglyceridemia				0.355
	Grade 1	1 (3%)	4 (13%)	
Hyperuricemia				1.000
	Grade 1	1 (3%)	2 (6%)	
Hypoalbuminemia				1.000
	Grade 1	0	1 (3%)	
Hypertension				0.581
	Grade 1	7 (24%)	9 (29%)	
	Grade 2	2 (7%)	4 (13%)	

## Data Availability

The data presented in the study are available in article.

## Supplementary Materials

The following are available online at https://www.mdpi.com/article/10.3390/nu13114022/s1, Table S1: Changes in other monitored parameters in the study.
