# Supplementary material for: Plant-Derived Lactobacillus paracasei IJH-SONE68 Improves Chronic Allergy Status: A Randomized, Double-Blind, Placebo-Controlled Clinical Trial
Source: Nutrients. 2021 Nov 11;13(11):4022. doi: 10.3390/nu13114022 (PMC8623948; doi:10.3390/nu13114022)
Supplement: Supplementary file 1 [file nutrients-13-04022-s001.zip › nutrients-1441395-supplementary.pdf]

**Supplementary Table S1.** Changes in other monitored parameters in the study.

| | IJH-SONE68 (*n* = 29) | Placebo (*n* = 31) | *p* value |
|---|---|---|---|
| Body weight (kg) | | | 0.937 |
| Baseline | 55.4 ± 2.9 | 56.4 ± 2.2 | |
| Change at 12 week | 0.16 ± 0.25 | 0.14 ± 0.25 | |
| Body fat (%) | | | 0.058 |
| | 24.7 ± 1.2 | 24.6 ± 1.7 | |
| | -0.14 ± 0.36 | 0.81 ± 0.35 | |
| Systolic blood pressure (mmHg) | | | 0.289 |
| | 110.8 ± 2.9 | 118.6 ± 3.0 | |
| | -0.60 ± 1.79 | 2.1 ± 1.7 | |
| Diastolic blood pressure (mmHg) | | | 0.196 |
| | 67.8 ± 2.1 | 73.5 ± 2.2 | |
| | -0.27 ± 1.28 | 2.1 ± 1.2 | |
| ALT (U/L) | | | 0.022 |
| | 18.4 ± 1.9 | 19.4 ± 1.3 | |
| | -2.4 ± 0.8 | 0.004 ± 0.747 | |
| Albumin (g/dL) | | | 0.766 |
| | 4.58 ± 0.06 | 4.41 ± 0.06 | |
| | -0.072 ± 0.039 | -0.055 ± 0.041 | |
| Alkaline phosphatase (U/L) | | | 0.020 |
| | 182.1 ± 11.2 | 213.5 ± 11.0 | |
| | -11.8 ± 3.9 | 0.75 ± 3.63 | |
| Amylase (U/L) | | | 0.656 |
| | 78.8 ± 3.5 | 80.8 ± 4.5 | |
| | 1.3 ± 1.7 | 2.3 ± 1.7 | |
| AST (U/L) | | | 0.001 |
| | 22.9 ± 1.4 | 22.7 ± 0.9 | |
| | -2.9 ± 0.5 | -0.46 ± 0.54 | |
| Total bilirubin (mg/dL) | | | 0.914 |
| | 0.76 ± 0.05 | 0.68 ± 0.05 | |
| | -0.023 ± 0.040 | -0.029 ± 0.045 | |
| Blood urea nitrogen (mg/dL) | | | 0.280 |
| | 14.0 ± 0.8 | 14.0 ± 0.6 | |
| | -0.43 ± 0.45 | 0.26 ± 0.45 | |
| LDL-cholesterol (mg/dL) | | | 0.969 |
| | 137.5 ± 6.6 | 132.7 ± 6.9 | |
| | -4.0 ± 3.4 | -3.9 ± 3.2 | |
| HDL-cholesterol (mg/dL) | | | 0.909 |
| | 76.1 ± 3.6 | 67.8 ± 3.0 | |
| | 2.2 ± 1.6 | 2.0 ± 1.5 | |
| Total-cholesterol (mg/dL) | | | 0.563 |
| | 228.6 ± 9.2 | 222.1 ± 6.8 | |
| | 0.07 ± 3.69 | -2.9 ± 3.6 | |
| Cholinesterase (U/L) | | | 0.034 |
| | 347.4 ± 19.2 | 321.7 ± 13.7 | |
| | -10.9 ± 4.4 | 2.0 ± 4.2 | |
| Creatinine (mg/dL) | | | 0.662 |
| | 0.67 ± 0.03 | 0.69 ± 0.03 | |
| | 0.001 ± 0.120 | 0.008 ± 0.120 | |
| Fasting blood glucose (mg/dL) | | | 0.471 |
| | 96.9 ± 1.8 | 99.3 ± 1.3 | |
| | 1.1 ± 1.5 | 2.6 ± 1.4 | |
| γ-GTP (U/L) | | | 0.109 |
| | 21.7 ± 3.5 | 28.7 ± 2.8 | |
| | -3.1 ± 1.2 | -0.29 ± 1.21 | |
| Lactate dehydrogenase (U/L) | | | 0.262 |
| | 188.6 ± 7.1 | 196.0 ± 6.9 | |
| | -9.9 ± 3.4 | -4.0 ± 3.2 | |
| Total protein (g/dL) | | | 0.801 |
| | 7.5 ± 0.1 | 7.3 ± 0.1 | |
| | -0.19 ± 0.08 | -0.16 ± 0.09 | |
| Triglyceride (mg/dL) | | | 0.941 |
| | 73.3 ± 7.3 | 103.5 ± 10.6 | |
| | -0.43 ± 7.53 | 0.36 ± 7.28 | |
| Ureic acid (mg/dL) | | | 0.498 |
| | 4.8 ± 0.3 | 4.9 ± 0.2 | |
| | -0.18 ± 0.11 | -0.072 ± 0.106 | |

Data are indicated as mean ± S.E.

*p* values are calculated by ANCOVA using each baseline value as a covariate.
